# The Putative Type III Secreted *Chlamydia abortus* Virulence-Associated Protein CAB063 Targets Lamin and Induces Apoptosis

**DOI:** 10.3389/fmicb.2020.01059

**Published:** 2020-05-25

**Authors:** Miriam Theresia Marschall, Ulrike Simnacher, Paul Walther, Andreas Essig, Jürgen Benjamin Hagemann

**Affiliations:** ^1^Institute of Medical Microbiology and Hygiene, Ulm University Hospital, Ulm, Germany; ^2^Central Facility for Electron Microscopy, Ulm University, Ulm, Germany

**Keywords:** *Chlamydia abortus*, CAB063, lamin, apoptosis, virulence

## Abstract

Since intracellular survival of all chlamydiae depends on the manipulation of the host cell through type III secreted effector proteins, their characterization is crucial for the understanding of chlamydial pathogenesis. We functionally characterized the putative type III secreted *Chlamydia abortus* protein CAB063, describe its intracellular localization and identified pro- and eukaryotic binding partners. Based on an experimental infection model and plasmid transfections, we investigated the subcellular localization of CAB063 by immunofluorescence microscopy, immunoelectron microscopy, and Western blot analysis. Pro- and eukaryotic targets were identified by co-immunofluorescence, co-immunoprecipitation, and mass spectrometry. Transmission electron microscopy and flow cytometry were used for morphological and functional investigations on host cell apoptosis. CAB063 localized in the nuclear membrane of the host cell nucleus and we identified the chaperone HSP70 and lamin A/C as pro- and eukaryotic targets, respectively. CAB063-dependent morphological alterations of the host cell nucleus correlated with increased apoptosis rates of infected and CAB063-transfected cells. We provide evidence that CAB063 is a chaperone-folded type III secreted *C. abortus* virulence factor that targets lamin thereby altering the host cell nuclear membrane structure. This process may be responsible for an increased apoptosis rate at the end of the chlamydial developmental cycle, at which CAB063 is physiologically expressed.

## Introduction

*Chlamydia* (*C.*) *abortus* is a zooanthroponotic pathogen common in ruminants (Essig and Longbottom, 2015), in which it causes enzootic abortions of ewes (EAE) and thus accounts for considerable economic damage (Longbottom and Coulter, 2003). Moreover, anecdotal evidence and the presence of antibodies in human sera suggest transmission to pregnant women and severe septic disease with miscarriage (Walder et al., 2005; Hagemann et al., 2016). The family of *Chlamydiaceae* has adapted to an obligate intracellular lifestyle with a unique biphasic developmental cycle (Elwell et al., 2016). As nutrients are acquired from the host cell, reduction of genome size (Sakharkar et al., 2004) and slimming of own synthetic pathways took place. However, this economization inevitably led to nutritional dependence on the host cell. It is therefore crucial for chlamydial survival to assure nutrient supply by modulation of the host cell metabolism. A well-known strategy of intracellular pathogens is the delivery of type III secreted effector proteins to the host cell cytosol, where they serve the purpose of virulence attainment and host cell manipulation (Cosse et al., 2018). Since these effectors have to be passed through the membrane of the intracellular compartment referred to as an inclusion, a sophisticated type III secretion needle apparatus is required (Nans et al., 2015b). It is pivotal for chlamydial pathogenicity (Wolf et al., 2006; Ur-Rehman et al., 2012), their uptake and survival (Nans et al., 2015a). Increasing evidence even suggests type III secretion system needle proteins to help confer protective immunity against chlamydial infections (Koroleva and Kobets, 2017; O’Meara et al., 2017). Our group provided ultrastructural evidence for the presence of a needle apparatus in *C. abortus* (Wilkat et al., 2014) and identified immunogenic putative virulence proteins (Forsbach-Birk et al., 2013; Hagemann et al., 2016). One of them, CAB063, was suggested to be type III secreted based on *in silico* analyses (Arnold et al., 2009) and its type III secreted orthologue, *Chlamydia psittaci* secreted inner nuclear membrane-associated Chlamydia protein (SinC), was shown to accumulate at the host cell nuclear membrane (Mojica et al., 2015), where it led to alterations in host cell nucleus integrity and function. Recent evidence also suggests a role of *Chlamydia caviae* SinC in virulence in an egg model (Filcek et al., 2019). We therefore aimed to investigate the subcellular localization of CAB063 in experimentally infected and plasmid-transfected HeLa cells and studied its influence on the host cell nucleus and host cell survival. The identification of pro- and eukaryotic binding partners helped to elucidate potential functions of CAB063 in chlamydial infections.

## Materials and Methods

### Organisms and Cell Culture for Experimental Infection

*Chlamydia abortus* S26/3 was grown in HeLa 229 cells as described previously (Forsbach-Birk et al., 2013). For experimental infection, inoculum was added with an MOI of 5 to semi-confluent HeLa cells (confluence of 70–80%). Depending on the research question posed, cells were processed for further work-up at 0, 24, or 48 h post-infection (hpi). Glass coverslips placed in the wells prior to infection served for fluorescence microscopy-based growth controls with an anti LPS^FITC^ antibody (Bio-Rad Laboratories GmbH, Munich, Germany).

Cloning experiments were carried out in *Escherichia coli* K12 DH5α that was cultured and selected on LB (lysogeny broth) agar plates or in LB broth with or without 100 μg/ml ampicillin.

### Transfection of HeLa Cells and Expression of Recombinant CAB063

Transfection was performed as described elsewhere (Forsbach-Birk et al., 2013). In short, *C. abortus* DNA was isolated and purified according to manufacturer instructions (QIAmp^®^ DNA Mini Kit, Qiagen GmbH, Hilden, Germany). Primers for PCR-based *CAB063* gene amplification were designed with CloneManager 7 (Scientific & Educational Software, Denver, United States), and read fwd 5′-AACGCTGGTACCGCAGTACGTCTTCTATGTC-3′ and rev 5′- ACCGGCGCTGAATTCTTAATCCTCTGACAC-3′. The pCI expression (Promega GmbH, Mannheim, Germany) and the peGFP vector were isolated from *E. coli* K12 DH5α cultures (QIAprep^®^ Spin Miniprep Kit, Qiagen GmbH, Hilden, Germany). Primers for EGFP amplification read fwd 5′-AAACGGTACCGCGGGCCCGG-3′ and rev 5′-AAAT CTAGAGTCGCGGCCGCTTTAC-3′. The DNA fragments and the expression vector were digested with EcoRI, KpnI, SaII, XbaI restriction enzymes (Roche Diagnostics Deutschland, GmbH, Mannheim, Germany), the resulting fragments were ligated (USB^®^ Ligate-IT^TM^ Rapid Ligation Kit, Affymetrix Inc., Santa Clara, United States) and transformed into competent *E. coli* K12 DH5α. Grown colonies were picked for overnight incubation in LB broth with ampicillin for further propagation and plasmid isolation was performed the following day. Successful cloning was checked via plasmid sequencing (Abi Prism 310 Genetic Analyzer, Applied Biosystems, Foster City, CA, United States).

For transfection, 1 × 10^5^ HeLa cells were seeded in each well of a twelve-well culture plate to achieve a semiconfluence of 70–80% the following day. Transient transfection was performed with a commercial reagent (jetPRIME^®^ *in vitro* DNA & siRNA transfection reagent, Polyplus-transfection SA, Illkirch, Germany) according to manufacturer instructions with 0.5 μg plasmid DNA. pCI-EGFP was used as a transfection control. Cells were incubated for 48 h at 37°C under 5% CO_2_ until they were further processed. Transfection efficiency was 13% as assessed by fluorescence microscopy and flow cytometry.

*Escherichia coli* containing a vector encoding His-tagged CAB063 was cultured in LB medium with ampicillin and protein expression was induced with 1 mM isopropyl-β-D-thiogalactopyranoside (IPTG) for 5 h at 37°C and 180 rpm. Bacteria were then centrifuged (10,000 rpm, 4°C, 10 min) and lyzed. Lysis and purification of His-tagged CAB063 was performed with a commercial kit (Protino^®^ Ni-TED 1000, Machery-Nagel GmbH & Co. KG, Düren, Germany) based on immobilized metal ion affinity chromatography (Yip and Hutchens, 1992).

### Direct Immunofluorescence for Subcellular Localization of CAB063

Immunofluorescence microscopy was performed as previously described (Forsbach-Birk et al., 2013). Briefly, HeLa cells grown on glass coverslips were either fixed with 99.8% methanol or 3.7% paraformaldehyde (for subsequent permeabilization with saponin) for 30 min at room temperature and pre-treated with 4% goat serum in PBS for 1.5 h to avoid unspecific binding prior to antibody treatment and microscopy. Anti LPS (Progen Biotechnik GmbH, Heidelberg, Germany) as well as rabbit-anti CAB063 antibodies that were generated in a previous work of our group (Forsbach-Birk et al., 2013) were used as primary antibodies for direct immunofluorescence microscopy (Axio Imager M2, Carl Zeiss Imaging Solutions, Jena, Germany). AlexaFluor^®^ 488-conjugated goat-anti rabbit IgG (H+L), highly cross-adsorbed (Invitrogen/Life Technologies GmbH, Darmstadt, Germany), were used as secondary antibodies diluted in Evan’s Blue-PBS solution that served as a DNA counter stain. Axio Vision 40 Software was used for imaging.

### Double Immunofluorescence for Identification of CAB063 Binding Partners

In addition to the staining of CAB063, mouse-anti lamin A/C was added and AlexaFluor^®^ 647-conjugated goat-anti mouse (Invitrogen/Life Technologies GmbH, Darmstadt, Germany) served as secondary antibodies to visualize co-localization of CAB063 and lamin A/C.

### Transmission (TEM) and Immunogold Electron Microscopy (IEM)

Electron microscopy served for detailed imaging of infected and transfected HeLa cells and was performed as described in a previous work of our group (Wilkat et al., 2014). In short, cells were grown, infected, or transfected in a 12-well culture plate fitted with UV-sterilized sapphire discs. Overgrown discs were removed for further workup after 24, 48, or 72 hpi. For TEM, samples were high-pressure frozen, freeze-substituted, embedded in epoxy resin in a three-step process, sliced in 70 nm thick sections, fixed on copper grids, and stained with lead citrate. For IEM, sapphire discs were worked up identically until freeze substitution: An osmium tetroxide-free substitution medium was used. Samples were washed with acetone twice, then embedded in three steps in LR-gold at −20°C, sliced in 90 nm thick slices, and mounted on copper grids. Unspecific binding sites were blocked with 4% goat serum. Samples were incubated with rabbit-anti CAB063 antibodies (1:50 in 4% goat serum, 30 min at room temperature). After washing, gold-labeled goat-anti rabbit secondary antibodies (Aurion, Wageningen, Netherlands) were added and incubated equally. After another washing, samples were fixed with 1% glutaraldehyde in PBS, washed with H_2_O, and post-stained with 1% uranyl acetate in H_2_O. TEM and IEM images (JEM-1400, JOEL GmbH, Eching, Germany; Acceleration voltage of 120kV) were assessed with iTEM Software (Olympus Soft Imaging Solutions GmbH, Münster, Germany).

### Co-immunoprecipitation With Anti CAB063 for the Identification of Possible Binding Partners of CAB063

Identification of CAB063 binding partners was performed by co-immunoprecipitation. Both experimentally infected and pCI-CAB063 transfected HeLa cells were lyzed and fractionated (whole cell lysate and nuclear fraction) according to manufacturer instructions using Proteo Extract^®^ Subcellular Proteome Extraction Kit (Merck KGaA, Darmstadt, Germany) and Qproteome Nuclear Protein Kit (Qiagen GmbH, Hilden, Germany) as described previously (Forsbach-Birk et al., 2013). An anti PARP antibody included in the kit was used to control fractionation and identify the nuclear fraction that was used for further experiments as such (Supplementary Figure S1). 60 μg of lysate were incubated with anti CAB063 overnight, while this and further incubation steps were conducted at 4°C to reduce unspecific binding. 20 μl of sepharose beads were added thereafter and incubated for 2 h. After centrifugation and washing to remove unbound components, the sepharose-protein pellet was solubilized in Lämmli (for 1D-SDS gels) or rehydration buffer (for 2D gel electrophoresis) and heated to remove the beads prior to electrophoresis. To maximize the yield of candidate proteins, selected nuclear fractions were enriched with 2 μg of recombinant CAB063 to literally fish for eukaryotic targets.

### Reverse Co-immunoprecipitation With Anti Lamin A/C for the Confirmation of CAB063 Binding

Immunoprecipitation was performed as described above. However, to test potential association of CAB063 with A-type lamins, anti lamin A/C antibodies were used to catch lamin A and thereby its binding partner CAB063.

### SDS-PAGE and Western Blotting for the Identification of Binding Partners

Lysates were separated by one- or two-dimensional gel electrophoresis. Two-dimensional electrophoresis (2D-SDS-PAGE) with isoelectric focusing in immobilized pH gradients was performed on Protean IEF Cell (Bio-Rad Laboratories, GmbH, Munich, Germany) according to manufacturer instructions. Silver-staining was done with a commercially available kit (FireSilver fixation kit, Proteome Factory AG, Berlin, Germany).

For Western blotting, lysates were separated by 1D-SDS-PAGE and were then blotted on a PVDF membrane. To reduce unspecific binding, membranes were incubated with blocking buffer at 4°C overnight. Incubation with rabbit-anti CAB063 (1:1,000 in TRIS buffered saline) or mouse-anti lamin A/C (Invitrogen/Life Technologies GmbH, Darmstadt, Germany; 1:1,000 in TRIS buffered saline) was also conducted at 4°C overnight, horseradish peroxidase labeled polycloncal goat-anti rabbit immunoglobulin (Dako, Glostrup, Denmark) was added the following day for 1 h at room temperature (1:1,000 in TRIS buffered saline with Tween). Antigen/antibody complexes were visualized on X-ray film with enhanced chemiluminescence solution (GE Healthcare, Munich, Germany).

Mass spectrometric identification of bands (1D-SDS-PAGE) and spots (2D-SDS-PAGE) was performed by Proteome Factory AG, Berlin, Germany, as previously described (Forsbach-Birk et al., 2013).

### Flow Cytometry for Analysis of Apoptosis and Cell Death

Flow cytometry served to measure cell death (propidium iodide, PI) and apoptosis (AnnexinV and fluorescent labeled inhibitors of caspase, FLICA) using the AnnexinV^APC^ Apoptose detection kit (BD Biosciences Europe, Darmstadt, Germany) and SR-Flica^®^ *in vitro* caspase detection kit (ImmunoChemistry Technologies LCC., Bloomington, United States). pCI-CAB063, pCI-EGFP transfected as well as non-transfected cells were harvested after trypsination and collected together with cell culture suspension. Following centrifugation, pellets were treated according to manufacturer instructions. Samples were analyzed with a FACScalibur^TM^ flow cytometer and FlowJo software (BD Biosciences Europe, Heidelberg, Germany).

### Statistical Analysis

Statistical analysis was performed with Microsoft Excel 2013 and GraphPad Prism 6.0. The Mann-Whitney U test was used to calculate the level of significance. Statistical significance was accepted at *p* ≤ 0.05.

## Results

### CAB063 Accumulates Along the Morphologically Altered Host Cell Nuclear Membrane

**FIGURE 1:**
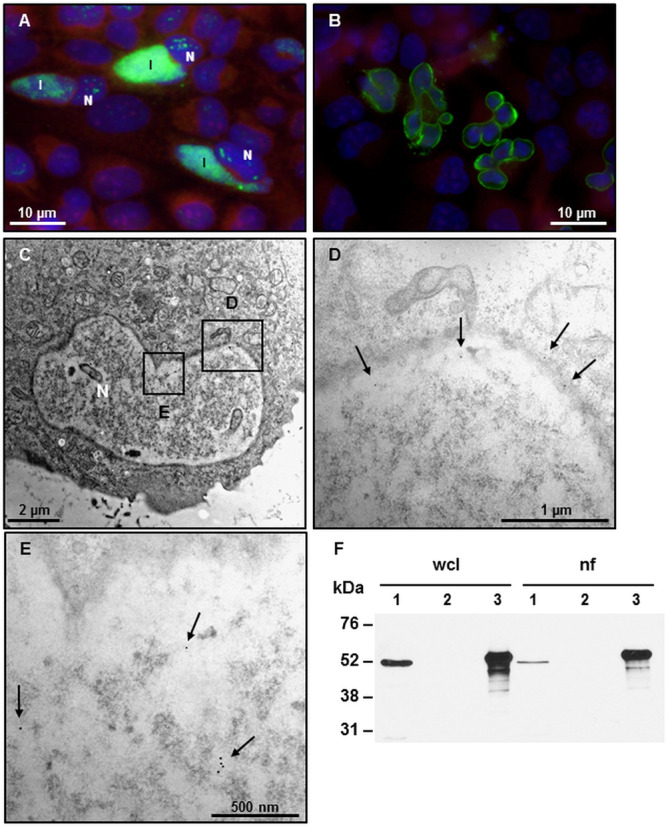
CAB063 accumulates along an altered nuclear membrane and in the nucleoplasm. **(A)** Direct immunofluorescence of experimentally infected HeLa cells at 48 h post-infection (hpi) shows localization of CAB063 both in the inclusion (I) and the host cell nucleus (N). **(B)** pCI-CAB063 transfected HeLa cells at 48 h post-transfection confirm a marked accumulation of CAB063 along the host cell nuclear membrane and reveal lobe-like morphological alterations of the nuclei in ≥85% of *n* = 50 investigated transfected cells. **(C)** 8000-fold magnification of a transfected HeLa cell with a lobulated nucleus. **(D)** 12,000-fold magnification of the section in panel **(A)** showing immunogold particles localizing in the nuclear membrane (arrows). **(E)** 14,000-fold magnification of the section in panel **(A)** showing immunogold particles localizing in the nucleoplasm of the host cell (arrows). In total, *n* = 10 cells were systematically investigated for immunogold staining. **(F)** CAB063 localizes in the nuclear fraction. Experimentally infected HeLa cells at 48 hpi (lane 1) and uninfected HeLa cells (lane 2) as well as recombinant CAB063 (lane 3) is shown for whole cell lysate (wcl) and the nuclear fraction (nf). CAB063 possesses a molecular weight of around 54 kDa. A representative Western blot using rabbit-anti CAB063 out of *n* = 3 independent experiments is provided.

In an effort to localize CAB063 more in detail, we transfected HeLa cells with pCI-CAB063. Thereby, we could confirm previous work in which we found CAB063 to localize both in the chlamydial inclusion and the host cell nucleus (Forsbach-Birk et al., 2013) (Figure 1A). Moreover, we observed a pronounced enrichment of CAB063 along the host cell nuclear membrane and lobe-like morphological alterations of nuclei in transfected cells (Figure 1B). These findings were verified by transmission electron microscopy (Figure 1C). Immunogold labeling revealed CAB063 localization along the nuclear membrane (Figure 1D) and within the nucleoplasm (Figure 1E). To confirm its presence in infected cells (Forsbach-Birk et al., 2013) and to substantiate its accumulation in the host cell nucleus, we performed Western blot analyses on whole cell lysates and nuclear fractions of experimentally infected HeLa cells (Figure 1F). The prominent accumulation of CAB063 along the nuclear membrane and the conspicuous morphological changes of the host cell nuclear shape were the basis for the further investigations on CAB063.

### CAB063 Associates With Chlamydial HSP70

**FIGURE 2:**
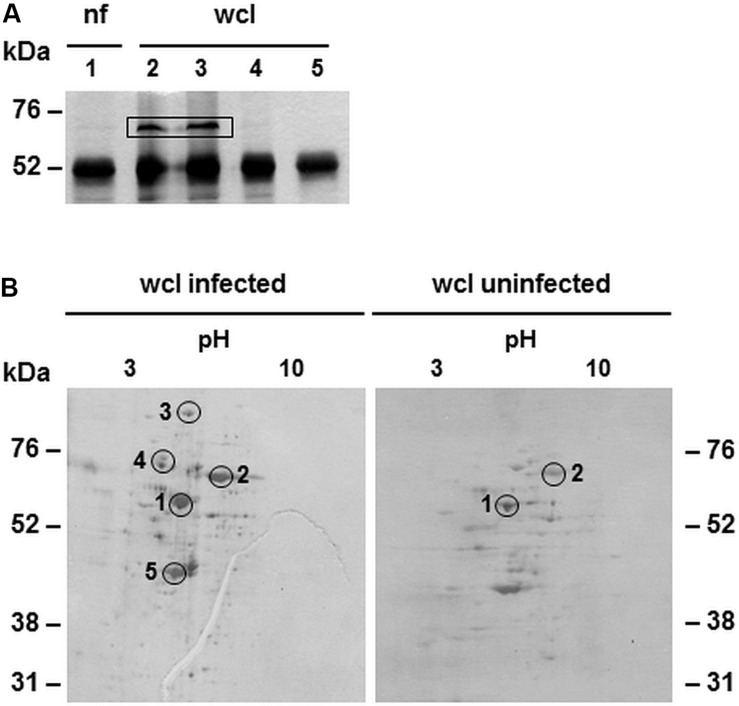
Binding partners of CAB063 in experimentally infected HeLa cells. **(A)** Silver staining of 1D-SDS-PAGE after co-immunoprecipitation; nuclear fraction (nf) of experimentally infected HeLa cells at 48 h post-infection (hpi) (lane 1), corresponding whole cell lysate (wcl, lanes 2 and 3), uninfected cells (lane 4), H_2_O negative control (lane 5). The prominent band of around 52 kDa equals anti CAB063 ± CAB063. Bands of 70 kDa are only present in infected HeLa cells and were mass spectrometrically identified as *C. abortus* chaperone DnaK (heat shock protein 70, HSP70). A representative silver staining out of *n* = 3 independent experiments is provided. **(B)** Silver staining of 2D-SDS-PAGEs after co-immunoprecipitation of whole cell lysates (wcl) of experimentally infected HeLa cells at 48 hpi showing several spots specific for infected cells. Spots 1 and 2 equal anti CAB063 ± CAB063 and the eukaryotic transketolase, respectively. Additional spots were mass spectrometrically identified as the chlamydial polymorphic membrane protein D (pmpD) (3), DnaK (chlamydial HSP70) (4), and elongation factor Tu (5). Representative silver stainings out of *n* = 3 independent experiments each are provided.

To identify possible binding partners of CAB063, we performed co-immunoprecipitation and 1D gel electrophoresis with subsequent silver staining of experimentally infected HeLa cells (Figure 2A). Prominent bands present in whole cell lysate but not the nuclear fraction of infected cells were mass spectrometrically identified as the chlamydial chaperone DnaK, a member of the prokaryotic heat shock protein 70 (HSP70) family. We confirmed these findings by 2D gel electrophoresis of whole cell lysates of experimentally infected HeLa cells compared to uninfected cells (Figure 2B). One of the specific spots only present in infected cells confirmed DnaK (HSP70) as a binding partner for CAB063.

### The Intermediate Filament Lamin Is a Main Eukaryotic Target of CAB063

**FIGURE 3:**
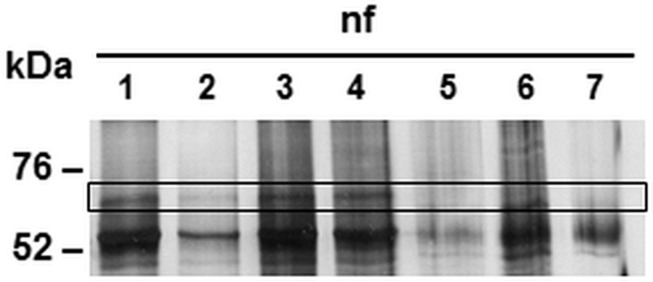
Binding partners of CAB063 in the nucleus of experimentally infected HeLa cells. Silver staining of 1D-SDS-PAGE after co-immunoprecipitation showing the nuclear fraction (nf) of HeLa cells. Fractions of experimentally infected cells at 48 h post-infection (lanes 1 and 2) and uninfected cells (lanes 3 and 4) were additionally enriched with recombinant CAB063. Remaining lanes show nuclear fractions of uninfected (lane 5) or experimentally infected cells at 48 hpi (lane 6) without additional recombinant CAB063, as well as an H_2_O negative control (lane 7). Human pre-lamin A/C was identified performing mass spectrometry on the 70 kDa band present in the nuclear fraction of infected and uninfected cells enriched with recombinant CAB063. A representative silver staining out of *n* = 3 independent experiments is provided.

**FIGURE 4:**
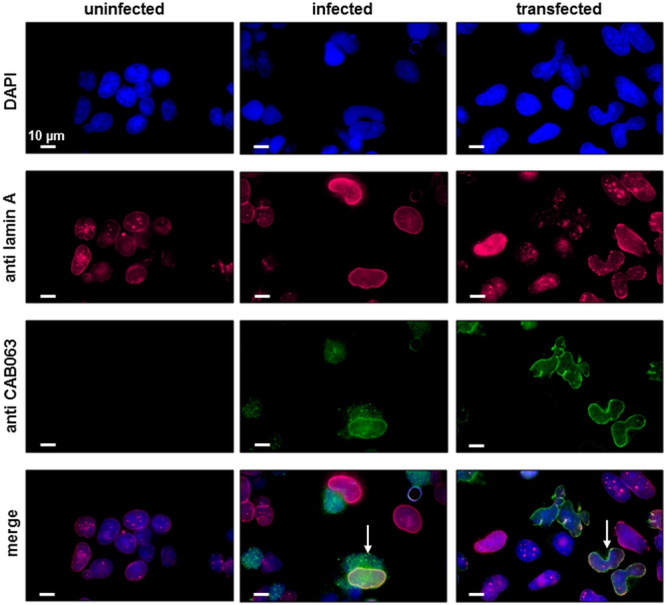
CAB063 co-localizes with lamin A along the host cell nuclear membrane. Co-localization (arrows) of CAB063 and lamin A is shown in pCI-CAB063 transfected and experimentally infected HeLa cells at 48 h post-infection and post-transfection, respectively. Goat-anti mouse AlexaFluor^®^ 647-conjugated secondary antibodies were used to visualize mouse-anti lamin A/C antibodies (pink), goat-anti rabbit AlexaFluor^®^ 488-conjugated secondary antibodies were used to visualize rabbit-anti CAB063 (green), DAPI was used for DNA staining (blue). Uninfected HeLa cells served as a control. Representative photographs out of *n* = 3 independent experiments are provided.

After the identification of a prokaryotic binding partner for CAB063, we aimed to identify the eukaryotic nucleus-associated target. Thus, we performed co-immunoprecipitation of CAB063 enriched nuclear fractions of uninfected and infected HeLa cells with subsequent 1D gel electrophoresis and silver staining (Figure 3). Visible bands at 70 kDa were excised and mass spectrometrically identified as pre-lamin A/C (Supplementary Table S1). Since fluorescence microscopy revealed a pronounced enrichment of CAB063 in the nuclear membrane, we hypothesized lamin A/C to be its primary binding partner. To test this possibility, we performed co-localization experiments with both pCI-CAB063 transfected and experimentally infected HeLa cells (Figure 4). Our experiments revealed co-localization of CAB063 and lamin A/C in a morphologically altered, lobulated nuclear membrane.

**FIGURE 5:**
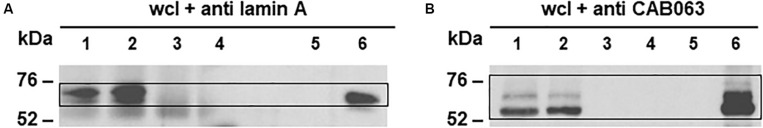
CAB063 binds lamin A. **(A)** Western blot with anti lamin A of a 1D-SDS-PAGE of HeLa whole cell lysates (wcl) after co-immunoprecipitation with anti CAB063. Marked bands at 70 kDa in experimentally infected (lane 1) and pCI-CAB063 transfected cells (lane 2) at 48 h post-infection or post-transfection, whereas uninfected cells (lane 3), H_2_O control (lane 4), and recombinant CAB063 (lane 5) show no bands. Recombinant lamin A with a size of 70 kDa served as a positive control (lane 6). **(B)** Western blot with anti CAB063 of a 1D-SDS-PAGE of HeLa whole cell lysates (wcl) after co-immunoprecipitation with anti lamin A. Marked bands at 54 kDa in experimentally infected (lane 1) and pCI-CAB063 transfected cells (lane 2), whereas uninfected cells (lane 3), H_2_O control (lane 4), and recombinant lamin A (lane 5) show no bands. Recombinant CAB063 with a size of 54 kDa served as a positive control (lane 6). Representative Western blots out of *n* = 3 independent experiments each are provided.

To verify specific binding of lamin A/C as the target of CAB063, we performed co-immunoprecipitation with whole cell lysates of pCI-CAB063 transfected as well as experimentally infected HeLa cells with subsequent Western blot analysis. Here, we first used anti CAB063 for co-immunoprecipitation and anti lamin A for Western blotting, while in a second reverse approach, we used anti lamin A for co-immunoprecipitation and anti CAB063 for Western blotting, respectively (Figure 5). The first approach expectedly revealed a protein band of around 70 kDa, correlating with the band of recombinant lamin A (Figure 5A). Accordingly, the second approach revealed prominent bands of around 54 kDa equivalent to the size of recombinant CAB063 (Figure 5B). In summary, these experiments revealed CAB063 association with A-type lamins, which are important nuclear intermediate filament proteins.

### CAB063-Dependent Nuclear Alterations Correlate With Increased Apoptosis

**FIGURE 6:**
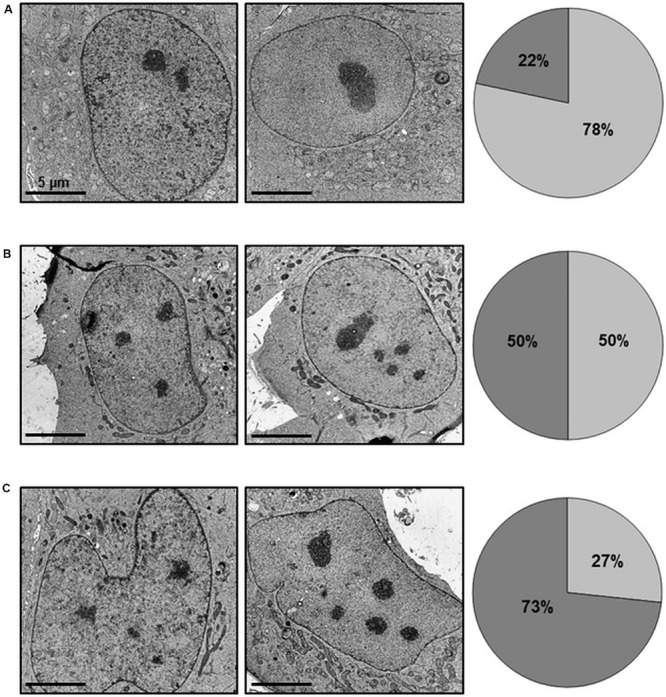
CAB063 causes morphological alterations in nuclei of transfected HeLa cells. 8,000-fold magnification of untreated **(A)**, pCI-GFP transfected **(B)** and pCI-CAB063 transfected **(C)** HeLa cells at 48 h post-transfection. Nucleoli number partly depends on transfection itself (increased number of nuclei with unchanged nuclear morphology), while an increased number of nuclei in addition to altered nuclear morphology is only observed in pCI-CAB063 transfection. Pie charts indicate the percentages of cells with ≤2 nucleoli (light gray) and ≥3 nucleoli (dark gray).

Since we observed characteristic lobe-like alterations in host cell nucleus morphology, we systematically characterized these changes and checked for apoptosis and cell death rates. To exclude the influence of transfection, we examined pCI-CAB063 and pCI-GFP transfected cells by electron microscopy. Untreated HeLa cells showed round to oval nuclei with mostly two or less nucleoli (Figure 6A), whereas transfection itself as controlled by pCI-GFP led to an increase in nucleoli number without any changes in nuclear architecture (Figure 6B). Transfection with pCI-CAB063 led to a further increase in nucleoli number and to characteristically lobulated nuclei as concordant with our primary fluorescence microscopic findings (Figures 1B, 6C). To exclude mere artifacts, we investigated experimentally infected HeLa cells as a control and could observe similar morphological nuclear changes by TEM (Supplementary Figure S2), albeit less prominent. Regarding apoptosis and cell death of HeLa cells at 48 hpi/pt (post-transfection), transfection itself did not markedly influence rates (Table 1). However, transfection with pCI-CAB063 significantly increased apoptosis from roughly 20 to 30% compared to the pCI-GFP control transfection (*p* ≤ 0.02). While around 20% of untreated HeLa cells were apoptotic or dead at 48h of incubation, their number increased to over 40% and over 50% after experimental infection, respectively (*p* ≤ 0.02 each) (Table 1).

**TABLE 1:** Apoptosis and cell death rates of infected and transfected HeLa 229 cells.

		*n*	hpi	Annexin V-positive	FLICA-positive	PI-positive
Negative control		5	0	9.60.6	9.10.5	12.41.4
	Untreated		24	13.11.7	12.71.6	11.51.8
			48	21.91.4	22.41.6	23.41.8
Transfected	pCI-GFP	8	48	19.71.7	20.41.6	16.32.6
	pCI-CAB063	8	48	29.92.2	31.92.9	17.92.8
Positive control		5	0	9.90.9	10.20.7	16.11.6
	Infected		24	13.21.2	13.11.3	14.71.8
			48	41.63.0	41.02.7	53.34.1

*Untreated HeLa control compared to vector control (pCI-GFP) and pCI-CAB063 transfected as well as to *C. abortus* S26/3 infected cells at different instances of time given as hours post-infection (hpi). Percentages are given as mean ± SEM.*

## Discussion

We investigated the putative virulence-associated protein CAB063 for its intracellular localization, its pro- and eukaryotic targets as well as its functional role in *C. abortus* infection.

We could confirm the localization of CAB063 in the chlamydial inclusion and the host cell nucleoplasm (Forsbach-Birk et al., 2013). Moreover, we could show in detail that CAB063 is particularly enriched along the nuclear membrane of both experimentally infected and CAB063-transfected cells. To our knowledge, CAB063 is currently the only *C. abortus* protein that was shown to accumulate in the host cell nucleus. Interestingly, several *Chlamydia trachomatis* proteins have been described to be delivered into the host cell nucleus as well (Hobolt-Pedersen et al., 2009; Lei et al., 2011, 2013) and the *C. psittaci* CAB063 orthologue SinC has been shown to accumulate along the host cell nuclear membrane of infected cells (Mojica et al., 2015). However, in contrast to SinC which was also found in neighboring uninfected cells, we could not observe similar effects for CAB063.

We showed lamin A/C, a type V intermediate filament of the A-type lamin family with crucial functions in nuclear architecture and stability, chromatin organization, signaling, and gene regulation (Gruenbaum and Foisner, 2015; Kim et al., 2017; de Leeuw et al., 2018; Perepelina et al., 2019), to be a eukaryotic target of CAB063. Here, the staining pattern of CAB063 both inside the nucleoplasm and along the nuclear membrane most likely reflects association to both soluble and membrane-associated lamin A/C portions. In contrast to B-type lamins which are tightly nuclear membrane-associated, A-type lamins are neither farnesylated nor carboxy-methylated and therefore more soluble and mobile, especially in interphase cells (Naetar et al., 2017). Details of the CAB063-lamin interplay, especially the exact differentiation of A-type lamins which was not possible based on our mass spectrometry data, as well as the identification of the CAB063 binding region, remain to be clarified in future investigations.

Present in both experimentally infected and CAB 063 transfected cells, we assume the lobulated changes in host cell nucleus architecture to be a consequence of the interference between CAB063 and the intermediate filament protein lamin. This assumption is corroborated by similar morphological changes in both sinC-transfected cells (Mojica et al., 2015) and cells in severe diseases caused by genetic mutations of the LMNA gene resulting in dysfunctional lamin A termed progerin (Goldman et al., 2004; Merideth et al., 2008). Especially pre-mature aging syndromes (progeria) (Kreienkamp and Gonzalo, 2019) and muscular dystrophies (Mio et al., 2019) substantiate the pivotal role of lamin A for cell regulation, host cell and nucleus integrity. In our study, transfected HeLa cells revealed more pronounced effects than did infected HeLa cells, suggesting either a different amount of CAB063 to be active in the two different models or further chlamydial proteins not present in the transfection model to modulate nucleotropic CAB063 effects.

Since CAB063 had been detected both in the inclusion and the host cell nucleus of infected cells, it had to be translocated to the nucleus. As indicated above, type III secretion systems are a common strategy for intracellular pathogens to release effector proteins from their intracellular niche into the cytosol and beyond. These are typically bound to mediator proteins like chaperones to facilitate folding or delivery. We hypothesized CAB063 to be type III secreted and to bind to a chaperone that would assist folding and subsequently facilitate transport through the inclusion membrane. *In silico* analyses suggested CAB063 to be type III secreted (Arnold et al., 2009) and our group provided morphological evidence for the presence of a type III secretion system in *C. abortus* (Wilkat et al., 2014). In agreement with these findings, we identified the chlamydial chaperone DnaK (heat shock protein 70, HSP70) as a prokaryotic target of CAB063. Since HSP70 chaperones are involved in protein folding (Bukau et al., 2000; Maleki et al., 2016) as well as translocation (Ryan and Pfanner, 2001; Stevens et al., 2003), our data provide further evidence for CAB063 to be a type III secreted chlamydial effector protein, as they occur in other *Chlamydia* species as well (Mojica et al., 2015; da Cunha et al., 2017; Wen et al., 2019).

Although CAB063 transcription could not be detected any earlier than 32 hpi and reached its maximum at 44 hpi, CAB063 protein could be found as early as 24 hpi in the chlamydial inclusions and from 36 hpi onward in the nuclear fraction of infected HeLA cell (Forsbach-Birk et al., 2013). Together with our observations, these findings offer different interpretations: i) CAB063 might be a preformed type III secreted effector of infectious elementary bodies that could support virulence by delivering early functions in the infection process. Interestingly, a recent study on the *C. caviae* CAB063 orthologue SinC provided first evidence that SinC was directly linked to virulence in an *in-vivo* model (Filcek et al., 2019). ii) CAB063 might play a regulatory role during and at the end of the developmental cycle which spans roughly 48 h. Targeting nuclear lamins could serve manifold regulatory effects beneficial for intracellular chlamydiae. However, depletion of lamin A has been shown to cause oxidative stress (Sieprath et al., 2015), which in turn based on mitochondrial damage causes apoptosis and cell death (Elmore, 2007) initiated and executed by caspases (Elmore, 2007; Green and Llambi, 2015). Indeed, we could show a significant increase in apoptosis in pCI-CAB063 transfected as well as in *C. abortus* infected HeLa cells. Interestingly, this effect was not measurable until 48 hpi consistent with the above mentioned kinetics of CAB063 expression (Forsbach-Birk et al., 2013).

In summary, we took advantage of the concerted use of fluorescence and immunogold transmission electron microscopy, co-immunoprecipitation as well as transfection and infection experiments to further characterize CAB063 as a likely type III secreted virulence-associated protein that targets HSP70 and influences host cell apoptosis by interfering with human lamin A/C at the host cell nuclear membrane.

## Data Availability Statement

All datasets generated for this study are included in the article.

## Author Contributions

AE and JH conceptualized the study. MM, US, and JH carried out the data curation, formal analysis, and investigations. AE, PW, MM, and JH did design of the experiments and methods. AE and JH supervised the whole project. JH wrote the original draft. AE, PW, MM, and US joined for reviewing and editing.

## Conflict of Interest

The authors declare that the research was conducted in the absence of any commercial or financial relationships that could be construed as a potential conflict of interest.
