# Supplementary material for: The Putative Type III Secreted Chlamydia abortus Virulence-Associated Protein CAB063 Targets Lamin and Induces Apoptosis
Source: Front Microbiol. 2020 May 25;11:1059. doi: 10.3389/fmicb.2020.01059 (PMC7261910; doi:10.3389/fmicb.2020.01059)
Supplement: Supplementary file 1 [file Table_1.docx]

**Table S1. Lamin A/C peptides identified by mass spectrometry.**

Mass spectrometry data based on MASCOT search performed by Proteome Factory AG, Berlin, Germany. Ions score is -10*Log(P), where P is the probability that the observed match is a random event. Individual ions scores >32 indicate identity or extensive homology (p<0.05). Protein scores are derived from ions scores as a non-probabilistic basis for ranking protein hits. Residues were identified and matched with uniprot (<https://www.uniprot.org/uniprot/P02545>).

| **residue** | **observed** | **mr (expt)** | **mr (calc)** | **ppm** | **miss** | **score** | **expect** | **peptide** |
| --- | --- | --- | --- | --- | --- | --- | --- | --- |
| 11 – 26 | 680.3482 | 1358.6818 | 1358.6790 | 2.02 | 0 | 94 | 5.2e-008 | R.SGAQASSTPLSPTR.I |
| 41 – 49 | 425.2455 | 848.4765 | 848.4756 | 1.05 | 0 | 45 | 0.0054 | R.LAVYIDR.V |
| 78 – 90 | 583.2788 | 1164.5411 | 1164.5411 | 1.74 | 0 | 87 | 2e-007 | K.AAYEAELGDAR.K |
| 240 – 250 | 514.7910 | 1027.5674 | 1027.5662 | 1.15 | 0 | 88 | 2.2e-007 | R.LADALQELR.A |
| 280 – 297 | 876.9362 | 1751.8579 | 1751.8550 | 1.61 | 0 | 112 | 9.3e-010 | R.NSNLVGAAHEELQQSR.I |
| 319 – 330 | 594.3230 | 1186.6314 | 1186.6306 | 0.68 | 1 | 26 | 0.54 | K.LRDLEDSLAR.E |
| 439 – 454 | 535.9427 | 1604.8063 | 1604.8046 | 1.08 | 1 | 45 | 0.0048 | R.VAVEEVDEEGKFVR.L |
| 527 – 542 | 746.3786 | 1490.7426 | 1490.7399 | 1.85 | 0 | 95 | 4.2e-008 | R.TALINSTGEEVAMR.K |
