# Supplementary figures and images for: The Putative Type III Secreted Chlamydia abortus Virulence-Associated Protein CAB063 Targets Lamin and Induces Apoptosis
Source: Front Microbiol. 2020 May 25;11:1059. doi: 10.3389/fmicb.2020.01059 (PMC7261910; doi:10.3389/fmicb.2020.01059)

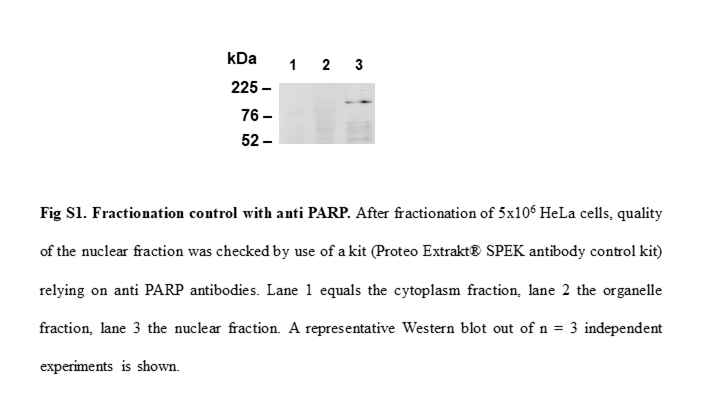

Supplement: Supplementary file 2 [file Image_1.TIF]

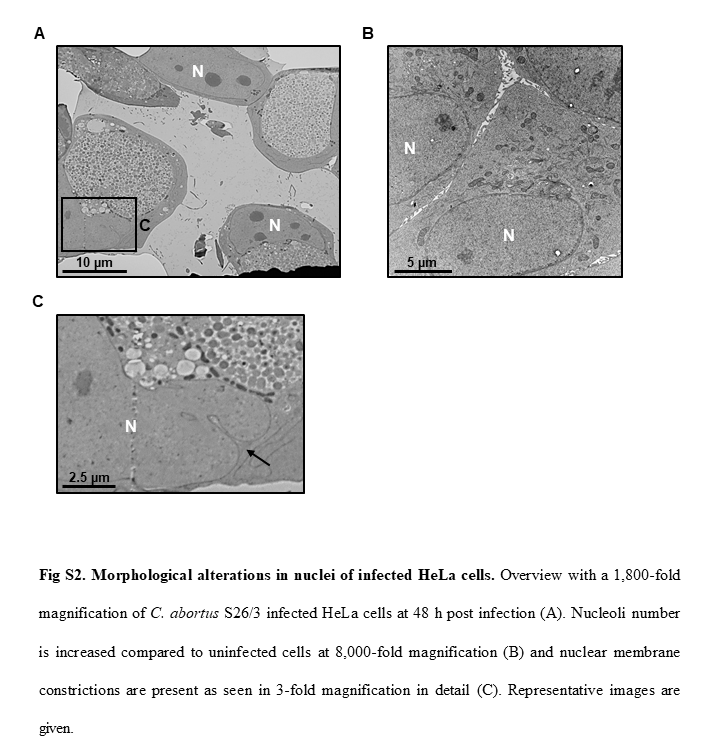

Supplement: Supplementary file 3 [file Image_2.TIF]
